# Smoking Behavior, Exposure to Second-Hand Smoke, and Attitudes Among Bulgarian and Foreign Medical Students

**DOI:** 10.3390/medsci13030134

**Published:** 2025-08-15

**Authors:** Dolina Gencheva Gencheva, Fedya Petrov Nikolov

**Affiliations:** 1 First Department of Internal Diseases, Medical University of Plovdiv, 66 “Peshtersko Shoes” Blv., 4002 Plovdiv, Bulgaria; 2 Clinic of Cardiology, University Multi-Profile Hospital for Active Treatment “Sveti Georgi”, 66 “Peshtersko Shoes” Blv., 4002 Plovdiv, Bulgaria

**Keywords:** smoking, cardiovascular risk, students, medical education, e-cigarettes, tobacco heating systems, heated tobacco products, cardiovascular diseases

## Abstract

**Background:** Cardiovascular morbidity and mortality are alarmingly high in Bulgaria, partly due to behavioral risk factors such as smoking. **Purpose:** This study aimed to assess and compare smoking habits, second-hand smoke exposure, and attitudes of Bulgarian and foreign medical students to better understand smoking behavior in this population. **Methods:** A cross-sectional survey was conducted among 1063 medical students at the Medical University of Plovdiv (60.8% women; 53% Bulgarian). **Results:** More Bulgarian students were active smokers and ever-smokers than foreign students (24.7% vs. 14% and 29.3% vs. 18.8%, *p* < 0.001). Bulgarian women smoked nearly as much as Bulgarian men (24.1% vs. 25.6% for active smokers, *p* > 0.05), whereas foreign women smoked less than foreign men (15.7% vs. 23.7%, *p* = 0.034). Women more often replaced classic cigarettes with tobacco heating systems (THSs) than men (40.7% vs. 25.3%, *p* = 0.020). Nearly 85% of the respondents started smoking by the age of 19. Exposure to second-hand smoke among friends, among colleagues, and in the family was associated with a higher risk of being an ever-smoker (ORs ~8.9; 3.4 and 3.7, respectively). About 20% of students were unsure or disagreed that smoking fewer cigarettes, THSs, or e-cigarettes posed health risks. The majority (61.3%) of active smokers acknowledged negative health effects. **Conclusions:** These findings highlight a concerning smoking prevalence among Bulgarian medical students and emphasize the need to strengthen medical education and health policies with updated tobacco risk information and targeted prevention programs to reduce smoking and improve future physicians’ cessation counseling skills. Smoking likely contributes significantly to Bulgaria’s high cardiovascular morbidity.

## 1. Introduction

Cardiovascular diseases (CVDs) remain a leading cause of morbidity and mortality worldwide. According to the World Health Organization (WHO), they are responsible for 17.9 million deaths annually, or 32% of deaths globally [1]. There are significant regional differences in CVD prevalence and mortality. The European Society of Cardiology classifies Bulgaria among regions with very high cardiovascular risk, with mortality rates exceeding 450/100,000 for men and 350/100,000 for women [2]. According to the National Statistical Institute (2024) [3], mortality in Bulgaria from circulatory system diseases (ICD codes I00-I99) reached 948.8/100,000 for men and 948.3/100,000 for women. Total mortality was 1677.0/100,000 for men and 1459.2/100,000 for women, with cardiovascular diseases accounting for 56.6% and 65% of all deaths, respectively. This recent data demonstrates the attenuation of the “protective effect” of the female sex in a country with a very high cardiovascular risk and, as far as mortality shares are concerned, its complete disappearance. Bulgaria also reports the highest CVD mortality rate in Europe [4]. Two conditions—myocardial infarction and stroke—account for the majority of CVD deaths. WHO data show they cause four out of five cardiovascular deaths [1]. In Bulgaria, similarly, the main causes are ischemic heart disease (222.0/100,000 men; 181.9/100,000 women) and cerebrovascular diseases (253.1/100,000 men; 289.9/100,000 women), representing 50% of cardiovascular mortality in both sexes [3].

Considering the gravity of the problem, it is crucial to examine the factors contributing to this unfavorable profile. Smoking is a major behavioral risk factor for cardiovascular diseases, causing over 8 million deaths annually, including 1.3 million from second-hand smoke, which represents the most prominent preventable contributor to death and disability worldwide [5]. The advertisement of alternative nicotine products poses additional challenges to public health efforts by perpetuating addiction without proven long-term risk reduction [6,7], and may introduce new dangers, such as those associated with e-cigarettes [8]. Tobacco use contributes to many non-communicable diseases, with a causal link to atherosclerotic cardiovascular disease, myocardial infarction, stroke, and peripheral artery disease, through mechanisms like endothelial damage, oxidative stress, and systemic inflammation [9,10].

Bulgaria faces serious smoking-related challenges. According to the OECD [11], 29% of adults smoked daily in 2019—the highest rate in the EU. In 2022, 32% of adolescentssmoked daily, also the highest rate in the EU. Smoking accounted for about 18% of all deaths in Bulgaria in 2019. Given these alarming trends, medical students are a key group to study. As future healthcare providers, they can help reduce public health risks through clinical work and advocacy, including delivering the internationally recommended “Very Brief Advice” on smoking [12]. Their own smoking behaviors and attitudes may affect how effectively they counsel patients and serve as role models. Assessing prevalence, exposure, and perceptions in this group is essential for developing targeted interventions.

**Purpose:** This study aimed to assess and compare smoking behaviors, second-hand smoke exposure, and perceptions of harm among Bulgarian and international medical students at The Medical University of Plovdiv in order to better understand smoking culture among young people and identify potential targets for intervention.

## 2. Materials and Methods

### 2.1. Study Design

We conducted a cross-sectional online survey directed at students currently studying medicine at the Medical University of Plovdiv, in Plovdiv, Bulgaria. The survey was performed using the Microsoft Forms platform (Microsoft Corporation, Albuquerque, NM, USA), with the possibility of accessing the questions only from an institutional account associated with the university, and the option “One response per person” was selected, ensuring participation of enrolled students and only one submission per account. The option for anonymous filling of the form was chosen with no names or e-mails provided to the research team, ensuring voluntary participation. The inclusion criteria for participation were as follows: being at least 18 years old, being a medical student at the Medical University of Plovdiv, and providing informed consent for the use of the submitted data for scientific purposes. Prior to accessing the questionnaire, participants were required to confirm that they met all inclusion criteria. Those who did not provide confirmation were excluded and unable to proceed further, as the survey could be completed only once per account. The study consisted of questions pertaining to the sociodemographic characteristics of the participants and of questions aimed at assessing lifestyle risk factors associated with cardiovascular diseases. The questionnaire was specifically developed by the authors for this study. While it has not undergone validation in prior research, it was carefully designed to address the study objectives. It included dichotomous closed-ended questions, multiple-choice questions, 5-point Likert scales for attitudes and perceptions, and open numerical fields. The dataset was screened for internal consistency (e.g., conflicting or implausible answers), with such entries excluded from analysis where applicable. The survey was promoted via the Microsoft Teams platform, and at lectures and classes attended by medical students, as well as by leaflets with a barcode at locations visited by medical students, such as libraries and other places on the university campus. Two identical but different-language (English and Bulgarian) versions of the survey were used, respectively, for the foreign and Bulgarian students. The sociodemographic questions and the questions about smoking are provided in Supplementary Material. The study was approved by the Ethics Committee of the Medical University of Plovdiv, Protocol N 3/21.03.2024.

### 2.2. Participants

The calculated sample size for achieving representability for the 2884 students currently studying medicine at the university, with a margin of error of 3% and confidence level of 95%, was 780 people [13]. A total of 1063 students completed the provided questionnaire between May and December 2024, well surpassing the aforementioned condition for representability. The response rate in our study was approximately 37%, which is above the average percentage reported by the National Survey of Student Engagement for online surveys among university students—28% [14].

### 2.3. Statistical Methods

Data were analyzed using IBM SPSS (version 27.0; SPSS, Inc., Chicago, IL, USA) and MedCalc Version 19.6.3 (MedCalc Software, Mariakerke, Belgium). Findings were considered statistically significant if *p* < 0.05. The following statistical methods were applied: Descriptive, Graphic, and Variation analyses, the Fisher–Freeman–Halton exact test, Fisher’s exact test and the Chi-squared test, the non-parametric tests of Kolmogorov–Smirnov and Shapiro–Wilk, the non-parametric test of Mann–Whitney, the independent-samples *t*-test of Student, and binary logistic regression analysis.

## 3. Results

### 3.1. Sociodemographic Characteristics of the Participants

Out of the 1063 students, 417 (39.2%) were men and 646 (60.8%) were women. The participants were divided into two main groups according to the answer to the question “Country of permanent residence”. Those who listed Bulgaria as a country of permanent residence (563 people or 53%) were referred to as Bulgarian students, and those who listed a different country as foreign students (n = 500; 47%). The mean age of the participants was 21.97 ± 2.49 years, ranging between 18 and 37 years. The Bulgarian and foreign students were statistically matched for the confounders age and sex (Table 1). The majority of the students who listed Bulgaria as their country of permanent residence declared themselves to be of Bulgarian ethnicity—85.3%—followed by Turkish and German ethnicities with 10.3% and 1.8%, respectively. The most common ethnicities listed by the foreign students were Pakistani (14.2%), followed by Greek (13.6%), and Italian (11.6%) (Supplementary Tables S1 and S2), and the most common countries of permanent residence were the United Kingdom (39%), Greece (13.4%), and Germany (12.6%) (Supplementary Table S3).

### 3.2. Smoking Status

Altogether 19.7% (n = 209) of the participants declared that they were active smokers (defined as a person who has smoked at least 100 cigarettes in their life and continues to smoke on most days), while 4.7% (n = 50) were former smokers (2% for at least one month, 1.0% for at least 6 months, and 1.7% for at least one year), making 24.4% ever-smokers. Significantly more Bulgarian students were active smokers compared to foreign students—24.7% vs. 14% (*p* < 0.001)—and ever-smokers—29.3% vs. 18.8%, with *p* < 0.001 (Table 2). When analyzed by sex (Supplementary Table S4), there was no significant difference between the male Bulgarian and foreign students despite the presence of a trend of more Bulgarian men being active smokers (25.6% vs. 17.5%, *p* > 0.05) and ever-smokers (30.5% vs. 23.7%, *p* = 0.125). Conversely, the proportion of active smokers and ever-smokers among Bulgarian women was approximately twice as high as that among foreign women (24.5% vs. 11.8%, *p* < 0.001, and 28.5% vs. 15.7%, *p* < 0.001, respectively). Additionally, there was a significantly higher share of foreign males who ever smoked vs. foreign women (23.7% and 15.7%, *p* = 0.034), but such a difference between the sexes was not present amongst the Bulgarian male and female students—in fact, the relative shares were almost equal (25.6% and 24.1%, *p* > 0.05 for active smokers; 30.5% and 28.5%, *p* > 0.05 for ever-smokers). In addition, among the foreign students, there was a significantly greater number of women who defined themselves as non-smokers compared to men (84.3% versus 76.3%, *p* = 0.026).

### 3.3. Cessation of Smoking

According to the ranking of responses (Supplementary Table S5) to the question available only to former smokers, “What made you stop smoking?”, the most common reason was “Concern about the occurrence of smoking-related diseases in the future” (68.0%), followed by “The unpleasant smell, changes in appearance” (32.0%), and “Occurrence of a health problem related to smoking” (26.0%). Interestingly, the least chosen reason was “Introduction of stricter measures/fines for smoking in public places or sanctions at workplaces” (2.0%). Comparative analysis between Bulgarian and foreign students revealed no significant differences in the reported motivations for smoking cessation (Supplementary Table S6). Among active smokers, those planning to quit significantly outnumbered those without such intentions—69.9% vs. 30.1% (*p* < 0.001). No statistically significant differences were found in cessation planning between the student groups or between sexes (Supplementary Tables S7 and S8).

### 3.4. Tobacco Products Used

**Figure 1:**
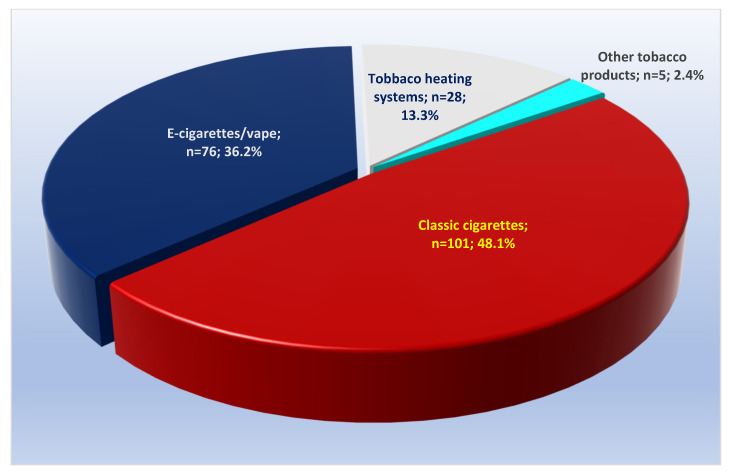
Frequency distribution of active smokers by the type of tobacco products used mostly at the time of the survey, showing that classic cigarettes represented less than 50% of all products.

When analyzed according to the answers to the question “What tobacco products do you mostly use at the moment?”, active smokers most often (48.1%) indicated classic cigarettes, followed by tobacco heating systems with 36.2% and electronic cigarettes/vape with 13.3%, and only 2.4% of the respondents indicated other methods of tobacco product use (Figure 1). There were no significant differences in the indicated main use of tobacco products in a comparative analysis between Bulgarian and foreign students (Supplementary Table S9). On the other hand, when the two sexes were analyzed, there were significantly more females in the entire sample who mostly used tobacco heating systems, which turned out to be almost twice as popular amongst them—at 44.9%, compared to 25.3% in men (*p* = 0.004). Conversely, the use of “Classic cigarettes” was more common among men—at 58.2%, compared to 40.7% of women, with *p* = 0.012 (Supplementary Table S10).

Regarding the question “What tobacco products have you used mostly in your life?”, the majority of respondents reported “Classic cigarettes” (67%), followed by “Tobacco heating systems” (21.5%), “Electronic cigarettes/vape” (12.4%), and others (2%). No significant differences were found between the two study groups or between sexes (Supplementary Tables S11 and S12). To the question “In recent years, have you permanently replaced classic cigarettes with other cigarettes?” (among active smokers who had ever smoked classic cigarettes), 44.4% responded negatively, and 33.3% had switched to THSs, 14.8% to e-cigarettes/vapes, 6.9% to both, and 0.5% to other products. No significant differences were observed between Bulgarian and foreign students (Supplementary Table S13). However, women more often replaced classic cigarettes with THSs (40.9% vs. 22.8% men, *p* = 0.009), while more men—especially Bulgarian men—reported no such replacement (55.7% of all men; 64.6% of Bulgarian men) (Supplementary Table S14).

### 3.5. Starting Age, Duration of Smoking, and Pack-Years

**Figure 2:**
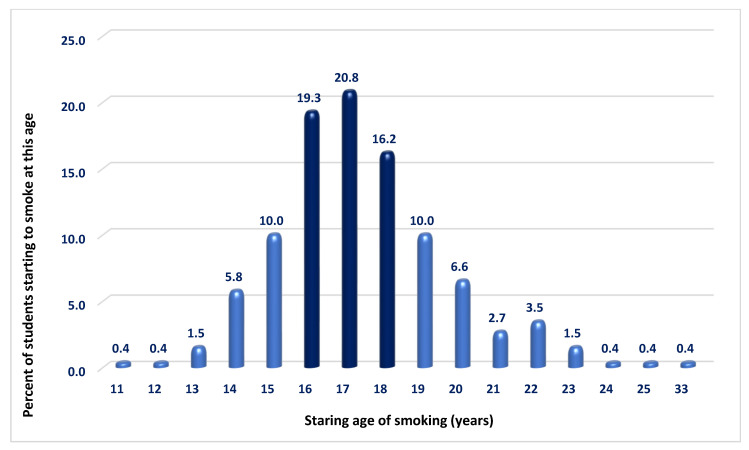
Frequency distribution of smoking initiation age. The highest frequencies of smoking initiation were observed at ages 16, 17, and 18, suggesting that most respondents began smoking before completing high school.

Among the 259 ever-smokers, the average age of smoking initiation was 17.38 ± 2.43 years (range 11–33 years). Smoking most often began at ages 17 (20.8%) and 16 (19.3%). About 18.1% started before age 15, and by age 17, the cumulative percentage rose to 58.3%. By ages 18 and 19—typically the age for completing school education —74.5% and 84.6% of the respondents had started smoking, respectively. Only about 15% initiated the habit after that age (Figure 2). No statistically significant differences were found between groups or sexes regarding initiation before or at ages 14, 16, or 18 (Supplementary Tables S15 and S16). The average duration of active smoking was 4.59 ± 2.82 years (range 1–15 years). Again, no significant differences were found between the two student groups or between sexes regarding the age of initiation or total duration of active smoking (Supplementary Table S17).

Given the known health impact of cigarette quantity, our study quantified usage among active smokers of classic cigarettes. Of them, 32.8% smoked up to 5 per day, 24.9% smoked 6–10, and 20.6% smoked 11–15, meaning that 92.1% consumed fewer than 20 cigarettes daily (i.e., less than one pack) (Supplementary Table S18). No statistically significant differences were found between the two student groups or sexes (Supplementary Tables S19 and S20).

### 3.6. Second-Hand Smoke in Social Circles and Influence on Smoking

Analysis of second-hand smoke exposure across three social circles—family, colleagues, and friends—showed that Bulgarian students significantly more often reported being “Always” or “Frequently” exposed than foreign students. Exposure for both groups of students was higher among friends (39.6% vs. 29.8%, *p* < 0.001) and colleagues (42.8% vs. 28.8%, *p* < 0.001), and lower within the family (25.2% vs. 13.2%, *p* < 0.001) (Table 3).

We examined the correlation between second-hand smoke exposure in key social settings and smoking status (Table 4). Ever-smokers significantly more often reported being “Frequently” (21.5%) and “Always” (11.6%) exposed to smoke in the family, compared to non-smokers (5.5% and 9.7%). Non-smokers more often answered “Never” or “Sometimes.” Similar patterns were observed for exposure among colleagues and friends, with ever-smokers reporting higher exposure and non-smokers more often indicating no exposure.

**Figure 3:**
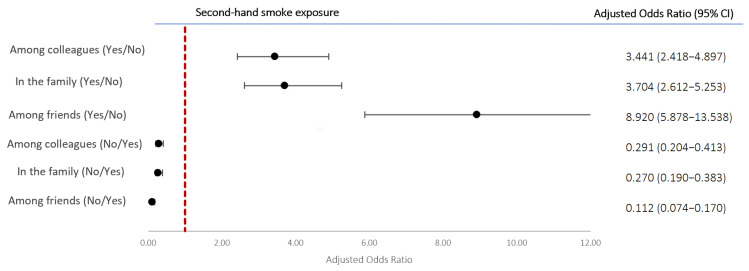
Odds ratios, adjusted for sex and age, and 95% CI of second-hand smoke as a factor associated with being an ever-smoker and its absence as a protective factor.

Binary logistic regression analysis, adjusted for age and sex, was performed to establish whether the presence of second-hand smoke in any of the three environments was a risk factor for being an ever-smoker. The respondents who declared being subjected “Frequently” or “Always” to second-hand smoke were considered exposed, while those who declared “Rarely” or “Never” were considered not exposed (Figure 3). Exposure among friends posed the highest risk—about nine times higher (aOR 8.920; 95% CI 5.878–13.538)—followed by exposure in the family (aOR 3.704; 95% CI 2.612–5.253) and among colleagues (aOR 3.441; 95% CI 2.418–4.897). Conversely, if the absence of exposure was viewed as a protective factor against taking up smoking, a lack of it in the family reduced the risk by 73%, a lack amongst colleagues by 71%, and a lack amongst friends by 89%.

### 3.7. Attitudes Toward Misconceptions of “Safe” Ways of Smoking and Associations with Smoking Status

In order to check what the medical students’ attitudes were regarding some common claims related to the existence of “safe” ways of smoking, we asked the following three questions: “To what extent do you agree with the statement that classic cigarettes, but in smaller quantities, are NOT associated with an increased health risk?”, “To what extent do you agree with the statement that tobacco heating systems are NOT associated with an increased health risk?”, and “To what extent do you agree with the statement that e-cigarettes/vapes are NOT associated with an increased health risk?”. For all three questions (Supplementary Table S21), the answers “Strongly disagree” and “Disagree” were the most frequently indicated ones, with their combined relative share reaching between 76.8 and 80.3% for the respective questions. It is also of interest, however, to note that for all three questions, the total share of medical students who either agreed or strongly agreed with the statements varied between 11.7% and 12.8%.

Altogether, the medical students who either believed that the aforementioned ways were not associated with health risks or could not assess if there was such a risk amounted to about 20% (19.8% for smaller quantities of classic cigarettes, 23.1% for THSs, and 20.4% for electronic cigarettes/vape). For the three questions, comparative analysis between the Bulgarian and the foreign students and between the two sexes was performed but revealed no significant differences (Supplementary Tables S23–S26 and S28), except in the use of electronic cigarettes/vape, where more male Bulgarian students answered “Strongly agree” compared to foreign males (9% vs. 4.1%, *p* = 0.046), while the foreign males more often answered “Disagree” compared to Bulgarian men (Supplementary Table S27).

### 3.8. Perceived Effects of Smoking on Health

In regard to the question “What effect do you believe smoking has exerted on your health?”, amongst the active smokers, the answer with the largest relative share (36.4%) was “Definitely negative”, followed by “Mostly Negative” (33.5%) and “ Neither negative nor positive” (24.9%), (Table 5). The comparative analysis did not find any significant differences between the two groups of students or between the two sexes (Supplementary Tables S29 and S30).

## 4. Discussion

Bulgaria has implemented the main elements of the WHO’s MPOWER strategy to reduce tobacco use [15]. Since 2012, smoking has been banned in enclosed public spaces, schools, workplaces, restaurants, bars, and public transport [16]. Despite these efforts, progress remains limited—smoking prevalence declined by just 0.3% between 2006 and 2014, compared to an average of 13.9% in the EU and 48.9% in Sweden [17]. In 2016, graphic warnings on cigarette packs became mandatory [18], and taxes on tobacco products, including THSs and e-cigarettes, have been gradually increased [19]. Although direct tobacco advertising is banned in major media, billboard and point-of-sale promotions remain legal [20].

Our findings show a higher share of active and ever-smokers among Bulgarian students compared to foreign peers, though the overall smoking rate was slightly lower than the OECD’s 2023 estimate of 29% [11]. This small difference may be explained by several factors, including differences in age groups surveyed, the influence of medical education on health behaviors, and a possible reporting bias due to social desirability among medical students. Unlike OECD data showing that men smoke nearly twice as much as women (38% vs. 21%), our study found similar rates: 25.6% in male and 24.5% in female Bulgarian students. This narrowing gender gap may signal a troubling trend of rising smoking rates among young women, potentially counteracting the traditionally protective effect of female sex against cardiovascular diseases and foreboding an even further rise in tobacco-related morbidity and mortality among Bulgarian women. This highlights the need for gender-sensitive smoking prevention and cessation strategies, especially targeting young female populations who may not traditionally be seen as high-risk groups for CVDs. Furthermore, the higher smoking rates among Bulgarian students support the view that Bulgaria’s high cardiovascular morbidity and mortality may be partly driven by modifiable behaviors, not just genetic or intrinsic factors.

Over two-thirds of active smokers in our study expressed an intention to quit, underscoring the importance of expanding support for cessation efforts. The majority of former smokers cited concern about future smoking-related diseases as their primary reason for quitting, highlighting the potential impact of health awareness campaigns. In contrast, only 2% reported that stricter public smoking regulations or workplace sanctions played a role. This suggests that personal motivation and internal acknowledgment of the harms might be much more crucial for cessation than external enforcement measures alone. These findings have important implications for tobacco control policy. While legislative actions such as bans and penalties remain essential for creating smoke-free environments, they may not be sufficient to drive meaningful behavioral change on their own. Therefore, future policies should place greater emphasis on strategies that foster intrinsic motivation, such as personalized risk communication, targeted health education campaigns, peer support interventions, and access to counseling services. Promoting self-efficacy and informed decision-making, rather than relying primarily on coercive approaches, may lead to more sustainable smoking cessation outcomes.

Another interesting point from our study is the decline in traditional cigarette use, which now accounts for a little less than half of all reported tobacco use among active smokers, alongside a rise in newer products such as THSs and e-cigarettes. Notably, replacing traditional cigarettes with tobacco heating systems was more common among women. Similar trends have been observed in Europe and the U.S., where novel tobacco products are marketed as less harmful alternatives. A systematic review by Sun et al. [21] (2015–2022) showed significant increases in THS use in both the European and Western Pacific regions, with higher usage among adolescents than adults (5.25% vs. 2.45%). The global market for THSs is projected to reach USD 49.14 billion in 2024, with an annual growth rate of 63.2% from 2025 to 2030 [22]. While these products are promoted as harm-reducing, their long-term health effects have not been assessed. E-cigarette use is also rising globally, largely driven by younger populations and supported by colorful marketing and flavored options—presenting a misleading modern solution to a centuries-old addiction issue [23]. Efforts to reduce tobacco use might be significantly undermined by its replacement with modified smoking habits amidst the health scare rather than quitting of smoking altogether.

The fact that the majority of students report that they had already initiated smoking between the ages of 15 and 19 is a strong argument for improving high school health education due to the highly addictive nature of the habit. This might necessitate more collaboration between educational institutions and the relevant health specialists, such as cardiologists and pulmonologists, in order to improve primary prevention. Addressing the situation at an early age may even be beneficial for the relatively new concept of primordial prevention [24]. In light of these findings, ministries of health and educational authorities should consider introducing structured collaboration with secondary schools to strengthen health education curricula before the anticipated age of smoking initiation. Furthermore, behavioral interventions shown to reduce smoking initiation in adolescents should be integrated into primary-care outreach and school-based programs. A systematic review conducted for the U.S. Preventive Services Task Force [25] found that behavioral interventions delivered in primary-care settings were effective in reducing the initiation of cigarette smoking among children and adolescents. Based on 13 randomized controlled trials (n = 21,700), such interventions led to a relative risk reduction of 18% in smoking initiation at 7 to 36 months of follow-up (RR = 0.82; 95% CI: 0.73–0.92) compared to minimal or no intervention. However, evidence for smoking cessation in adolescents who were already smokers was limited and not statistically significant, and pharmacological approaches did not show a clear benefit. These findings support the primordial prevention strategy by highlighting the role of early, non-pharmacological behavioral interventions in delaying or preventing the onset of tobacco use in youth populations.

Our data indicate that exposure to second-hand smoke is strongly linked to smoking behavior, with the highest influence observed among friends, followed by family and workplace environments. This suggests a reinforcing social dynamic that sustains the habit. Public campaigns could address the role of parental smoking in youth initiation, while stricter workplace regulations may help reduce smoking-related norms among colleagues. Although limited, prior research supports these patterns. Racic et al. (2014) reported that adolescents in Bosnia and Herzegovina were six times more likely to smoke if their parents did, and five times more likely if their friends did [26]. A study in China similarly linked second-hand smoke at home and in public places with increased smoking risk among adolescents [27].

Our study, focused on future healthcare professionals, provides insight into how personal health behaviors may influence preventive counseling. While most students disagreed with statements suggesting that certain forms of smoking (fewer classic cigarettes, THSs, or e-cigarettes) are safe, it is concerning that about 20% either agreed with their safety or were uncertain. Current smokers were significantly more likely to view these alternatives as safe or remain neutral, which may affect their future cessation advice to patients. This underscores the need for improved medical education that communicates that no form of tobacco or nicotine use is risk-free. Given physicians’ influence as health educators, integrating robust, up-to-date tobacco-related content into curricula is essential. Curricular reforms should address misconceptions, promote evidence-based understanding, and strengthen students’ skills in preventive counseling to improve future smoking cessation outcomes. To the best of our knowledge, this is the first study in Bulgaria to assess medical students’ attitudes toward the aforementioned ways of smoking.

Finally, most active smokers in our survey acknowledged a negative impact of smoking on their health, underscoring nicotine’s addictive nature and the challenge of quitting despite perceived harm. Interestingly, one third reported no noticeable effect, which may reflect the difficulty of abandoning habits that do not produce immediate or tangible health consequences.

**Limitations:** While the cross-sectional design that we used is well-suited for assessing prevalence and associations at a specific point in time, it inherently comes with certain limitations. One major constraint is the inability to establish causal relationships between variables, as data are collected at a single time point rather than over a period. Additionally, selection bias may arise, particularly in online surveys, where participation is voluntary and may favor certain groups, in this case, more motivated or health-conscious individuals. There is also the risk of information bias due to self-reporting, which can affect the accuracy of responses—especially for sensitive behaviors such as smoking. Lastly, the generalizability of the findings may be limited, and the sample might not be fully representative of the broader student population.

## 5. Conclusions

Rates of smoking among Bulgarian medical students were higher than those among foreign students, with a practically equal frequency between Bulgarian male and female respondents. Novel smoking methods such as tobacco heating systems and e-cigarettes accounted for slightly more than 50% of all uses among active smokers, with tobacco heating systems being significantly more popular with women. The initiation of smoking predominantly happened during high school age. Exposure to second-hand smoke in social environments raised the likelihood of being an ever-smoker. Smoking status significantly affected the attitudes toward perceived “safe” ways of smoking. These findings highlight the need to strengthen medical education with accurate, up-to-date content on tobacco risks. Tailored prevention and education programs for medical students could reduce use and improve their future role in smoking cessation counseling. Furthermore, addressing second-hand smoke exposure in youth environments is a critical public health priority, as it contributes significantly to smoking initiation and long-term health risks.

## Figures and Tables

**Table 1:** Sociodemographic characteristics of the participants in the survey.

Characteristics	Bulgarian Students (n = 563)		Foreign Students (n = 500)		*p*
	$\bar{X}$	SD	$\bar{X}$	SD	
Age (years)	21.85	2.39	22.12	2.59	0.055
Sex	n	**%**	n	**%**	
Male	223	39.6	194	38.8	0.801
Female	340	60.4	306	61.2	
**University** Year					0.007
I	114	20.2	96	19.2	0.683
II	100	17.8	60	12.0	0.008
III	51	9.1	73	14.6	0.005
IV	173	30.7	139	27.8	0.300
V	63	11.2	62	12.4	0.545
VI	62	11.0	70	14.0	0.139

**Table 2:** Comparative analysis of the two groups of students according to the answers to the question “Are you an active smoker?”.

Group	Answers	Bulgarian Students		Foreign Students		*p*
		n	%	n	%	
All (n = 1063)	Yes	139	24.7	70	14.0	<0.001
	No	398	70.7	406	81.2	<0.001
	Former smoker for at least 1 month	10	1.8	11	2.2	0.641
	Former smoker for at least 6 months	7	1.2	4	0.8	0.516
	Former smoker for at least 1 year	9	1.6	9	1.8	0.801
	Ever-smokers	165	29.3	94	18.8	**<0.001**
Men (n = 417)	Yes	57	25.6	34	17.5	0.295
	No	155	69.5	148	76.3	
	Former smoker for at least 1 month	5	2.2	4	2.1	
	Former smoker for at least 6 months	2	0.9	4	2.1	
	Former smoker for at least 1 year	4	1.8	4	2.1	
	Ever-smokers	68	30.5	46	23.7	0.125
Women (n = 646)	Yes	82	24.1	36	11.8	<0.001
	No	243	71.5	258	84.3	<0.001
	Former smoker for at least 1 month	5	1.5	7	2.3	0.455
	Former smoker for at least 6 months	5	1.5	0	0.0	0.032
	Former smoker for at least 1 year	5	1.5	5	1.6	0.918
	Ever-smokers	97	28.5	48	15.7	<0.001

**Table 3:** Comparative analysis of the two studied groups by exposure to second-hand smoke (answers “Always” and “Frequently”) *.

Environment	Bulgarian Students (n = 563)		Foreign Students (n = 500)		*p*
	n	%	n	%	
Family	142	25.2 ^a^	66	13.2 ^a^	<0.001
Colleagues	241	42.8 ^b^	140	28.0 ^b^	<0.001
Friends	223	39.6 ^b^	149	29.8 ^b^	<0.001

* Same letters in the columns designate a lack of a statistically significant difference, while different letters designate the presence of such a difference (*p* < 0.05); the *p*-values given in the table indicate comparisons in the rows.

**Table 4:** Analysis of the relationship between how often the respondents were exposed to second-hand smoke and whether they were ever-smokers (*p* < 0.001).

Environment	Answers	Ever-Smokers (n = 259)		Non-Smokers (n = 804)		*p*
		n	%	n	%	
Family	Always	30	11.6	44	5.5	<0.001
	Frequently	56	21.6	78	9.7	<0.001
	Sometimes	55	21.2	100	12.4	<0.001
	Rarely	33	12.7	123	15.3	0.304
	Never	85	32.8	459	57.1	<0.001
Colleagues	Always	52	20.1	69	8.6	<0.001
	Frequently	86	33.2	174	21.6	<0.001
	Sometimes	64	24.7	203	25.2	0.872
	Rarely	44	17.0	177	22.0	0.085
	Never	13	5.0	181	22.5	<0.001
Friends	Always	66	25.5	50	6.2	<0.001
	Frequently	96	37.1	160	19.9	<0.001
	Sometimes	65	25.1	207	25.7	0.847
	Rarely	26	10.0	181	22.5	<0.001
	Never	6	2.3	206	25.6	<0.001

**Table 5:** Frequency distribution of the answers to the question “What effect do you believe smoking has exerted on your health?” (analyzed for active smokers only).

Answers	n	%	Sp
Definitely negative	76	36.4	3.3
Mostly negative	70	33.5	3.3
Neither negative nor positive	52	24.9	3.0
Mostly positive	10	4.8	1.5
Definitely positive	1	0.5	0.5
Number of active smokers who responded	209		

## Data Availability

The data presented in this study are available on request from the corresponding author due to privacy reasons.

## Supplementary Materials

The following supporting information can be downloaded at https://www.mdpi.com/article/10.3390/medsci13030134/s1.

## Abbreviations

The following abbreviations are used in this manuscript:

CI	Confidence Interval
CVDs	Cardiovascular Diseases
EU	European Union
ICD	International Classification of Diseases
OECD	Organization for Economic Co-operation and Development
aOR	Adjusted Odds Ratio
THS	Tobacco Heating System
WHO	World Health Organization
