# Supplementary material for: Smoking Behavior, Exposure to Second-Hand Smoke, and Attitudes Among Bulgarian and Foreign Medical Students
Source: Med Sci (Basel). 2025 Aug 15;13(3):134. doi: 10.3390/medsci13030134 (PMC12372101; doi:10.3390/medsci13030134)
Supplement: Supplementary file 1 [file medsci-13-00134-s001.zip › medsci-3801417-supplementary.pdf]

SUPPLEMENTARY MATERIAL:

I. Questions used in the survey

A. Questions related to the socio-demographic characteristics of the participants

B. Questions related to smoking:

II. Supplementary tables

I. **Questions used in the survey:**

A. Questions related to the socio-demographic characteristics of the participants*:

* Available to all respondents:

1. Sex (Single choice)

1) Male

2) Female

2. Age (Single line text, the value must be a number)

3. Country of permanent residence (Single choice)

1) Bulgaria

2) Greece

3) Türkiye

4) The United Kingdom

5) North Macedonia

6) The United States of America

7) Ukraine

8) Italy

9) Germany

10) Afghanistan

11) Albania

12) Armenia

13) Australia

14) Azerbaijan

15) Bahrein

16) Bangladesh

17) Belarus

18) Belgium

19) Bhutan

20) Brunei Darussalam

21) Burundi

22) Canada
23) China
24) Congo
25) Cypris
26) Denmark
27) Egypt
28) Ethiopia
29) Finland
30) France
31) Gambia
32) Ghana
33) Hong Kong
34) Hungary
35) India
36) Iran
37) Iraq
38) Ireland
39) Israel
40) Japan
41) Jordan
42) Kazakhstan
43) Kenia
44) Kosovo
45) Kuwait
46) Latvia
47) Lebanon
48) Libya
49) Lithuania
50) Malaysia
51) Malta
52) Mexico
53) Moldova
54) Morocco
55) Nepal
56) Nigeria

57) Norway

58) Oman

59) Pakistan

60) Poland

61) Qatar

62) Russia

63) Santa Lucia

64) Saudi Arabia

65) Serbia

66) Sierra Leone

67) Singapore

68) Slovakia

69) Slovenia

70) Somalia

71) South Africa

72) South Korea

73) Spain

74) Sri Lanka

75) Sudan

76) Sweden

77) Switzerland

78) Syria

79) Tanzania

80) Thailand

81) The Netherlands

82) The Philippines

83) The United Arab Emirates

84) Tunisia

85) Uzbekistan

86) Yemen

87) Zambia

88) Zimbabwe

89) Other

4. Ethnicity* (Multiple choice)

**Kindly keep in mind that not all possible ethnicities corresponding to the countries from the previous question are listed as significant heterogenicity exists in some countries. If you do not find the ethnicity that best describes you, or believe you can provide a more specific answer, please describe in the option "Others".*

1) Albanian
2) Arab
3) Armenian
4) Australian
5) Bahraini
6) Belarusian
7) Bengalis
8) Bulgarian
9) Canadian
10) Chinese
11) Cypriot
12) Danish
13) Dutch
14) Egyptian
15) English
16) Ethiopian
17) Filipino
18) Finnish
19) French
20) German
21) Greek
22) Hungarian
23) Indian
24) Irish
60) Italian
25) Japanese
26) Jewish
27) Kazakh
28) Kurdish
29) Latvian
30) Lebanese

31) Lithuanian

32) Malays

33) Maltese

34) Mexican

35) Moldovan

36) North Macedonian

37) Norwegian

38) Oman

39) Persian

40) Polish

41) Qatari

42) Roma/Romani people

43) Russian

44) Scottish

45) Serbs

46) Slovak

47) Slovenes

48) Somali

49) South Korean

50) Spanish

51) Sri-Lankan/Sinhalese

52) Swede

53) Swiss

54) Thai

55) Turkish

56) Ukrainian

57) Uzbek

58) Welsh

59) Prefer not to say

60) Other

5. What year are you a medical student at MU - Plovdiv? (Single choice)

1) 1

2) 2

3) 3

4) 4

5) 5

6) 6

B. Questions related to smoking:

1. Are you an active smoker*? (Single choice)

**An active smoker is someone who has smoked at least 100 cigarettes in their lifetime and continues to smoke on most days*

*** Available to all respondents*

1) Yes

2) No

3) Former smoker of at least 1 month

4) Former smoker of at least 6 month

5) Former smoker of at least 1 year

2. What made you stop smoking? (Multiple choice) *

** Available to former smokers only*

1) Occurrence of a health problem related to smoking

2) Concern about the occurrence of smoking-related diseases in the future

3) Introduction of stricter measures/fines for smoking in public places or sanctions at workplaces

4) There was no specific reason

5) The unpleasant smell, changes in appearance

6) Consideration of other people

7) Pregnancy/breast-feeding

8) Change of environment

9) Feeling of dependence

10) Other

3. Do you plan to stop smoking? (Single choice) *

**Available to active smokers only*

1) Yes

2) No

4. What tobacco products do you mostly use at the moment? * (Single choice)

**Available to active smokers only*

1) Classic cigarettes

2) Electronic cigarettes/vape

3) Tobacco heating systems

4) Other

5. What tobacco products have you used mostly in your life? *

**Available to active and former smokers only*

1) Classic cigarettes

2) Electronic cigarettes/vape

3) Tobacco heating systems

4) Other

6. In recent years, have you permanently replaced classic cigarettes with other cigarettes? * (Single choice)

**Available to active smokers only*

1) No

2) Yes, with electronic cigarettes/vape

3) Yes, with tobacco heating systems

4) Yes, with both electronic cigarettes and tobacco heating systems

5) Other

7. At what age did you start smoking? * (Single line text, the value must be a number)

**Available to active and former smokers only*

8. How many years have you smoked actively? * (Single line text, the value must be a number)

**Available to active and former smokers only*

9. If you currently smoke or have smoked classic cigarettes, how many cigarettes per day on average? * (Single choice)

**Available to active smokers and former smokers only*

1) up to 5

2) 6 to 10

3) 11 to 15

4) 16 to 20

5) 21 to 25

6) 26 to 30

7) 31 to 35

8) 36 to 40

9) above 40

10) I have never smoked classic cigarettes

10. What effect do you believe smoking has exerted on your health? *

**Available to active smokers and former smokers only*

The effects is:

1) Definitely negative
2) Mostly negative
3) Neither negative nor positive
4) Mostly positive
5) Definitely positive

11. Are you exposed to passive* smoking? **

** Please consider only the situations where you are exposed to inhalation of air exhaled by smokers of classic cigarettes and/or tobacco heating systems, but not electronic cigarettes/vape; second-hand smoke.*

*** Available to all respondents*

In the family:

1) Always
2) Frequently
3) Sometimes
4) Rarely
5) Never

Among colleagues:

1) Always
2) Frequently
3) Sometimes
4) Rarely
5) Never

Among Friends:

1) Always
2) Frequently
3) Sometimes

4) Rarely
5) Never

12. To what extent do you agree with the statement that the following are NOT associated with an increased health risk?*

** Available to all respondents*

Classic cigarettes, but in smaller quantities:

1) Strongly agree
2) Agree
3) Neither agree nor disagree
4) Disagree
5) Strongly disagree

Tobacco heating systems:

1) Strongly agree
2) Agree
3) Neither agree nor disagree
4) Disagree
5) Strongly disagree

Electronic cigarettes/vape:

1) Strongly agree
2) Agree
3) Neither agree nor disagree
4) Disagree
5) Strongly disagree

II. Supplementary tables:

Supplementary Table S1: Frequency distribution of Bulgarian students by ethnicity

Ethnicity	n	%	Sp
Bulgarian	480	85.3	1.5
Turkish	58	10.3	1.3
German	10	1.8	0.6
Prefer not to say	7	1.2	0.5
Greek	4	0.7	0.4
English	3	0.5	0.3
Ukrainian	2	0.4	0.3
Italian	2	0.4	0.3
Arab	1	0.2	0.2
Armenian	1	0.2	0.2
Canadian	1	0.2	0.2
Filipino	1	0.2	0.2
French	1	0.2	0.2
Indian	1	0.2	0.2
Jewish	1	0.2	0.2
North Macedonian	1	0.2	0.2
Serbs	1	0.2	0.2
Pakistani	1	0.2	0.2
Nigerian	1	0.2	0.2

* The sum of the percentages exceeds 100, as some of the students declared more than one ethnicity
** - The percentage is calculated based on 563 Bulgarian students

Supplementary Table S2: Frequency distribution of foreign students by ethnicity

Ethnicity	n	%	Sp
Pakistani	71	14.2	1.6
Greek	68	13.6	1.5
Italian	58	11.6	1.4
Indian	52	10.4	1.4
German	47	9.4	1.3
Bulgarian	23	4.6	0.9
English	21	4.2	0.9
Somali	20	4.0	0.9
Turkish	19	3.8	0.9
Arab	18	3.6	0.8
Nigerian	13	2.6	0.7
Prefer not to say	12	2.4	0.7
Bengalis	11	2.2	0.7
North Macedonian	11	2.2	0.7
Cypriot	10	2.0	0.6
Sri-Lankan/Sinhalese	10	2.0	0.6
Nepali	10	2.0	0.6
Irish	9	1.8	0.6
Russian	7	1.4	0.5
Persian	6	1.2	0.5

British	5	1.0	0.4
Kurdish	4	0.8	0.4
Serbs	3	0.6	0.3
Swede	3	0.6	0.3
Afghan	3	0.6	0.3
African	3	0.6	0.3
Ghanaian	3	0.6	0.3
Chinese	2	0.4	0.3
Egyptian	2	0.4	0.3
Spanish	2	0.4	0.3
Ukrainian	2	0.4	0.3
Syrian	2	0.4	0.3
Sudanese	2	0.4	0.3
Zimbabwean	2	0.4	0.3
Albanian	1	0.2	0.2
Armenian	1	0.2	0.2
Australian	1	0.2	0.2
Danish	1	0.2	0.2
Dutch	1	0.2	0.2
Filipino	1	0.2	0.2
French	1	0.2	0.2
Japanese	1	0.2	0.2
Lebanese	1	0.2	0.2
Malays	1	0.2	0.2
Mexican	1	0.2	0.2
Scottish	1	0.2	0.2
Pontic Greek	1	0.2	0.2
American	1	0.2	0.2
South African	1	0.2	0.2
Asian	1	0.2	0.2
Assyrian	1	0.2	0.2
Baloch	1	0.2	0.2
British Asian	1	0.2	0.2
Colombiano	1	0.2	0.2
Ivorian	1	0.2	0.2
Kashmir	1	0.2	0.2
Mixed	1	0.2	0.2
Jordanese	1	0.2	0.2
North African	1	0.2	0.2
Portuguese	1	0.2	0.2
Saint Lucian	1	0.2	0.2
Sierra Leonean	1	0.2	0.2
Sri Lankan Tamil	1	0.2	0.2
Tanzanian	1	0.2	0.2
Tajik	1	0.2	0.2
Pashtun	1	0.2	0.2

* The sum of the percentages exceeds 100, as some of the students declared more than one ethnicity
** - The percentage is calculated based on 500 foreign students

Supplementary Table S3: Frequency distribution of the foreign students by country of permanent residence

Country of permanent residence	n	%	Sp
The United Kingdom	195	39.0	2.2
Greece	67	13.4	1.5
Germany	63	12.6	1.5
Italy	58	11.6	1.4
Ireland	30	6.0	1.1
North Macedonia	11	2.2	0.7
Turkiye	9	1.8	0.6
Cypris	7	1.4	0.5
Qatar	5	1.0	0.4
Sweden	5	1.0	0.4
Pakistan	4	0.8	0.4
Serbia	4	0.8	0.4
The United States of America	3	0.6	0.3
Canada	3	0.6	0.3
Denmark	3	0.6	0.3
Nigeria	3	0.6	0.3
Norway	3	0.6	0.3
Albania	2	0.4	0.3
Finland	2	0.4	0.3
Ghana	2	0.4	0.3
India	2	0.4	0.3
Spain	2	0.4	0.3
Australia	1	0.2	0.2
Bahrein	1	0.2	0.2
Belgium	1	0.2	0.2
Japan	1	0.2	0.2
Jordan	1	0.2	0.2
Kenia	1	0.2	0.2
Moldova	1	0.2	0.2
Morocco	1	0.2	0.2
Nepal	1	0.2	0.2
Santa Lucia	1	0.2	0.2
Syria	1	0.2	0.2
Palestine	1	0.2	0.2
The United Arab Emirates	1	0.2	0.2
Zimbabwe	1	0.2	0.2
Austria	1	0.2	0.2
Colombia	1	0.2	0.2
Tanzania	1	0.2	0.2
All	100	100.0	

Supplementary Table S4: Comparative analysis of the two sexes according to the answers to the question "Are you an active smoker?

Group	Answers	Men		Women		P
		n	%	n	%	
All (n=1063)	Yes	91	21.8	118	18.3	0.406
	No	303	72.7	501	77.6	
	Former smoker for at least 1 month	9	2.2	12	1.9	
	Former smoker for at least 6 months	6	1.4	5	0.8	
	Former smoker for at least 1 year	8	1.9	10	1.5	
	Ever-smokers	114	27.3	145	22.4	0.079
Bulgarian students (n=563)	Yes	57	25.6	82	24.1	0.900
	No	155	69.5	243	71.5	
	Former smoker for at least 1 month	5	2.2	5	1.5	
	Former smoker for at least 6 months	2	0.9	5	1.5	
	Former smoker for at least 1 year	4	1.8	5	1.5	
	Ever-smokers	68	30.5	97	28.5	0.637
Foreign students (n=500)	Yes	34	17.5	36	11.8	0.074
	No	148	76.3	258	84.3	**0.026**
	Former smoker for at least 1 month	4	2.1	7	2.3	0.883
	Former smoker for at least 6 months	4	2.1	0	0.0	**0.011**
	Former smoker for at least 1 year	4	2.1	5	1.6	0.682
	Ever-smokers	46	23.7	48	15.7	**0.034**

Supplementary Table S5: Frequency distribution of the answers to the question "What made you stop smoking?" (former smokers only)

Answers	n	%	Sp
Concern about the occurrence of smoking-related diseases in the future	34	68.0	6.6
Unpleasant smell, changes in appearance	16	32.0	6.6
Occurrence of a health problem related to smoking	13	26.0	6.2

Consideration of other people	12	24.0	6.0
Feelings of dependence	10	20.0	5.7
Change of environment	7	14.0	4.9
No particular reason	6	12.0	4.6
Other	3	6.0	3.4
Introducing stricter measures/fines for smoking in public places or workplace sanctions	1	2.0	2.0
**Number of respondents**	50		

*The sum of the percentages exceeds 100, as some of the respondents indicated more than one answer

Supplementary Table S6: Comparative analysis of the two studied groups according to the answers to the question "What made you stop smoking?" (former smokers only)

Answers	Bulgarian students (n=26)		Foreign students (n=24)		P
	n	%	n	%	
Occurrence of a health problem related to smoking	5	19.2	8	33.3	0.261
Concern about the occurrence of smoking-related diseases in the future	18	69.2	16	66.7	0.851
Introducing stricter measures/fines for smoking in public places or workplace sanctions	0	0.0	1	4.2	0.296
No particular reason	1	3.8	5	20.8	0.067
Unpleasant smell. changes in appearance	7	26.9	9	37.5	0.427
Consideration of other people	4	15.4	8	33.3	0.143
Pregnancy/breastfeeding	0	0.0	0	0.0	-
Change of environment	2	7.7	5	20.8	0.187
Feelings of dependence	12	46.2	7	29.2	0.221
Others	2	7.7	1	4.2	0.607

*The sum of the percentages exceeds 100, as some of the respondents indicated more than one answer

Supplementary Table S7: Comparative analysis of the two studied groups according to the answers to the question "Do you plan to stop smoking?"

Group	Do you plan to stop smoking?	Bulgarian students		Foreign students		P
		n	%	n	%	
All (n=210)	Yes	98	70.5	48	68.6	0.873
	No	41	29.5	22	31.4	
Men (n=92)	Yes	39	68.4	22	64.7	1.000
	No	18	31.6	12	35.3	
Women (n=118)	Yes	59	72.0	26	72.2	0.819
	No	23	28.0	10	27.8	

Supplementary Table S8: Comparative analysis of the two sexes according to the answers to the question "Do you plan to stop smoking?"

Group	Do you plan to stop smoking?	Men		Women		P
		n	%	n	%	
All (n=209)	Yes	61	67.0	85	72.0	0.451
	No	30	33.0	33	28.0	
Bulgarian students (n=139)	Yes	39	68.4	59	72.0	0.707
	No	18	31.6	23	28.0	
Foreign students (n=146)	Yes	22	64.7	26	72.2	0.609
	No	12	35.3	10	27.8	

Supplementary Table S9: Comparative analysis of the two groups of students according to the answers to the question "What tobacco products do you mainly use at the moment?"

Group	Answers	Bulgarian students		Foreign students		P
		n	%	n	%	
All (n=209)	Classic cigarettes	65	46.8	36	51.4	0.235
	E-cigarettes/vape	21	15.1	7	10.0	
	Tobacco heating systems	52	37.4	24	34.3	
	Others	1	0.7	3	4.3	
Men (n=91)	Classic cigarettes	33	57.9	20	58.8	0.382
	E-cigarettes/vape	9	15.8	4	11.8	
	Tobacco heating systems	15	26.3	8	23.5	
	Others	0	0.0	2	5.9	
Women (n=118)	Classic cigarettes	32	39.0	16	44.4	0.652
	E-cigarettes/vape	12	14.6	3	8.3	
	Tobacco heating systems	37	45.1	16	44.4	
	Others	1	1.2	1	2.8	

Supplementary Table S10 Comparative analysis of the two sexes according to the answers to the question "What tobacco products do you mainly use at the moment?"

Group	Answers	Men		Women		P
		n	%	n	%	
All (n=209)	Classic cigarettes	53	58.2	48	40.7	**0.012**
	E-cigarettes/vape	13	14.3	15	12.7	0.737
	Tobacco heating systems	23	25.3	53	44.9	**0.004**
	Others	2	2.2	2	1.7	0.794
Bulgarian students (n=139)	Classic cigarettes	33	57.9	32	39.0	0.063
	E-cigarettes/vape	9	15.8	12	14.6	
	Tobacco heating systems	15	26.3	37	45.1	
	Others	0	0.0	1	1.2	
Foreign students (n=70)	Classic cigarettes	20	58.8	16	44.4	0.328
	E-cigarettes/vape	4	11.8	3	8.3	
	Tobacco heating systems	8	23.5	16	44.4	
	Others	2	5.9	1	2.8	

Supplementary Table S11: Comparative analysis of the two groups of students according to the answers to the question “What tobacco products have you used mostly in your life?”

Group	Answers	Bulgarian students		Foreign students		P
		n	%	n	%	
All (n=209)	Classic cigarettes	91	65.5	49	70.0	0.515
	E-cigarettes/vape	20	14.4	6	8.6	0.232
	Tobacco heating systems	30	21.6	15	21.4	0.974
	Others	2	1.4	0	0.0	0.321
Men (n=91)	Classic cigarettes	37	64.9	24	70.6	0.578
	E-cigarettes/vape	9	15.8	4	11.8	0.800
	Tobacco heating systems	14	24.6	6	17.6	0.438
	Others	1	1.8	0	0.0	0.434
Women (n=118)	Classic cigarettes	54	65.9	25	69.4	0.711
	E-cigarettes/vape	11	13.4	2	5.6	0.245
	Tobacco heating systems	16	19.5	9	25.0	0.503
	Others	1	1.2	0	0.0	0.511

Supplementary Table S12: Comparative analysis of the two sexes according to the answers to the question "What tobacco products have you used mostly in your life?"

Group	Answers	Men		Women		P
		n	%	n	%	
All (n=209)	Classic cigarettes	61	67.0	79	66.9	0.988
	E-cigarettes/vape	13	14.3	13	11.0	0.475
	Tobacco heating systems	20	22.0	25	21.2	0.889
	Others	1	1.1	1	0.8	0.823
Bulgarian students (n=139)	Classic cigarettes	37	64.9	54	65.9	0.893
	E-cigarettes/vape	9	15.8	11	13.4	0.903
	Tobacco heating systems	14	24.6	16	19.5	0.474
	Others	1	1.8	1	1.2	0.772
Foreign students (n=70)	Classic cigarettes	24	70.6	25	69.4	0.913
	E-cigarettes/vape	4	11.8	2	5.6	0.359
	Tobacco heating systems	6	17.6	9	25.0	0.454
	Others	0	0.0	0	0.0	-

Supplementary Table S13: Comparative analysis of the two groups of students according to the answers to the question "In recent years, have you permanently replaced classic cigarettes with other cigarettes?"*

**analyzed among 189 active smokers who ever smoked classic cigarettes*

Group	Answers	Bulgarian students		Foreign students		P
		n	%	n	%	
All (n=189)	No	57	46.0	27	41.5	0.685
	Yes, with e-cigarettes/vape	17	13.7	11	16.9	
	Yes, with tobacco heating systems	42	33.9	21	32.3	
	Yes, simultaneously with e-cigarettes and tobacco heating systems	8	6.5	5	7.7	
	Yes, with others	0	0.0	1	1.5	
Men (n=79)	No	31	64.6	13	41.9	0.202
	Yes, with e-cigarettes/vape	4	8.3	5	16.1	
	Yes, with tobacco heating systems	10	20.8	8	25.8	
	Yes, simultaneously with e-cigarettes and tobacco heating systems	3	6.3	4	12.9	
	Yes, with others	0	0.0	1	3.2	
Women (n=110)	No	26	34.2	14	41.2	0.865
	Yes, with e-cigarettes/vape	13	17.1	6	17.6	
	Yes, with tobacco heating systems	32	42.1	13	38.2	
	Yes, simultaneously with e-cigarettes and tobacco heating systems	5	6.6	1	2.9	
	Yes, with others	0	0.0	0	0.0	

Supplementary Table S14: Comparative analysis of the two sexes according to the answers to the question "In recent years, have you permanently replaced classic cigarettes with other cigarettes?"*

**analyzed among 189 active smokers who ever smoked classic cigarettes*

Group	Answers	Men		Women		P
		n	%	n	%	
All (n=189)	No	44	55.7	40	36.4	**0.009**
	Yes, with e-cigarettes/vape	9	11.4	19	17.3	0.262
	Yes, with tobacco heating systems	18	22.8	45	40.9	**0.009**
	Yes, simultaneously with e-cigarettes and tobacco heating systems	7	8.9	6	5.5	0.365
	Yes, with others	1	1.3	0	0.0	0.232
Bulgarian students (n=124)	No	31	64.6	26	34.2	**0.001**
	Yes, with e-cigarettes/vape	4	8.3	13	17.1	0.167
	Yes, with tobacco heating systems	10	20.8	32	42.1	**0.015**
	Yes, simultaneously with e-cigarettes and tobacco heating systems	3	6.3	5	6.6	0.948
	Yes, with others	0	0.0	0	0.0	-
Foreign students (n=65)	No	13	41.9	14	41.2	0.409
	Yes, with e-cigarettes/vape	5	16.1	6	17.6	
	Yes, with tobacco heating systems	8	25.8	13	38.2	
	Yes, simultaneously with e-cigarettes and tobacco heating systems	4	12.9	1	2.9	
	Yes, with others	1	3.2	0	0.0	

Supplementary Table S15: Comparative analysis of the two groups of students, according to age of smoking initiation at or before 14, 16, and 18 years of age

Group	Starting age of smoking (years, at or before)	Bulgarian students		Foreign students		P
		n	%	n	%	
All (n=259)	14	13	7.9	8	8.5	1.000
	16	60	36.4	37	39.4	0.689
	18	123	74.5	70	74.5	1.000
Men (n=114)	14	4	5.9	5	10.9	0.481
	16	22	32.4	18	39.1	0.549
	18	46	67.6	34	73.9	0.535
Women (n=145)	14	9	9.3	3	6.3	0.751
	16	38	39.2	19	39.6	1.000
	18	77	79.4	36	75.0	0.671

Supplementary Table S16: Comparative analysis of the two sexes, according to age of smoking initiation at or before 14, 16, and 18 years of age

Group	Starting age of smoking (years, at or before)	Men		Women		P
		n	%	n	%	
All (n=259)	14	9	7.9	12	8.3	1.000
	16	40	35.1	57	39.3	0.519
	18	80	70.2	113	77.9	0.196
Bulgarian students (n=165)	14	4	5.9	9	9.3	0.562
	16	22	32.4	38	39.2	0.413
	18	46	67.6	77	79.4	0.104
Foreign students (n=94)	14	5	10.9	3	6.3	0.481
	16	18	39.1	19	39.6	1.000
	18	34	73.9	36	75.0	1.000

Supplementary Table S17: Comparative analysis of the two groups of students according to the answers to the questions "At what age did you start smoking?" and "How many years have you smoked actively?"

Question	Group	Bulgarian students			Foreign students			P
		n	$\overline{X}$	SD	n	$\overline{X}$	SD	
At what age did you start smoking?	All	165	17.33	2.47	94	17.47	2.38	0.938
	Men	68	17.74	2.92	46	17.52	2.48	0.517
	Women	97	17.05	2.06	48	17.42	2.30	0.571
	P=		0.067			0.842		
How many years have you smoked actively?	All	139	4.44	2.62	70	4.90	3.18	0.413
	Men	57	4.07	2.31	34	5.12	3.81	0.327
	Women	82	4.70	2.80	36	4.69	2.48	0.848
	P=		0.241			0.967		

Supplementary Table S18: Frequency distribution of the answers to the question "If you currently smoke or have smoked classic cigarettes, how many cigarettes per day on average?"

Answers	n	%	Sp
1 to 5	62	32.8	3.4
6 to 10	47	24.9	3.1
11 to 15	39	20.6	2.9
16 to 20	26	13.8	2.5
21 to 25	7	3.7	1.4
26 to 30	7	3.7	1.4
Above 40	1	0.5	0.5
31 to 35	0	0.0	
36 to 40	0	0.0	
**Number of respondents who smoked classic cigarettes**	189		

Supplementary Table S19: Comparative analysis of the two groups of students according to the answers to the question "If you currently smoke or have smoked classic cigarettes, how many cigarettes per day on average?"

Group	Answers	Bulgarian students		Foreign students		P
		n	%	n	%	
All (n=189)	1 to 5	40	32.3	22	33.8	0.991
	6 to 10	30	24.2	17	26.2	
	11 to 15	27	21.8	12	18.5	
	16 to 20	17	13.7	9	13.8	
	21+	10	8.1	5	7.7	
Men (n=79)	1 to 5	12	25.0	11	35.5	0.631
	6 to 10	10	20.8	4	12.9	
	11 to 15	12	25.0	8	25.8	
	16 to 20	7	14.6	6	19.4	
	21+	7	14.6	2	6.5	
Women (n=110)	1 to 5	28	36.8	11	32.4	0.493
	6 to 10	20	26.3	13	38.2	
	11 to 15	15	19.7	4	11.8	
	16 to 20	10	13.2	3	8.8	
	21+	3	3.9	3	8.8	

Supplementary Table S20: Comparative analysis of the two sexes according to the answers to the question "If you currently smoke or have smoked classic cigarettes, how many cigarettes per day on average?"

Group	Answers	Men		Women		P
		n	%	n	%	
All (n=189)	1 to 5	23	29.1	39	35.5	0.109
	6 to 10	14	17.7	33	30.0	
	11 to 15	20	25.3	19	17.3	
	16 to 20	13	16.5	13	11.8	
	21+	9	11.4	6	5.5	
Bulgarian students (n=124)	1 to 5	12	25.0	28	36.8	0.195
	6 to 10	10	20.8	20	26.3	
	11 to 15	12	25.0	15	19.7	
	16 to 20	7	14.6	10	13.2	
	21+	7	14.6	3	3.9	
Foreign students (n=65)	1 to 5	11	35.5	11	32.4	0.125
	6 to 10	4	12.9	13	38.2	
	11 to 15	8	25.8	4	11.8	
	16 to 20	6	19.4	3	8.8	
	21+	2	6.5	3	8.8	

Supplementary Table S21: Frequency distribution of answers to the following three questions: "To what extent do you agree with the statement that Classic cigarettes but in smaller quantities are NOT associated with an increased health risk?", "To what extent do you agree with the statement that Tobacco heating systems are NOT associated with an increased health risk?" and "To what extent do you agree with the statement that E-cigarettes/vapes are NOT associated with an increased health risk?"

Questions	Answers	n	%	Sp
To what extent do you agree with the statement that classic cigarettes, but in smaller quantities, are NOT associated with an increased health risk?	Strongly agree	613	57.7	1.5
	Agree	240	22.6	1.3
	Neither agree nor disagree	74	7.0	0.8
	Disagree	70	6.6	0.8
	Strongly disagree	66	6.2	0.7
	**Total number of respondents**	1063	100.0	
To what extent do you agree with the statement that Tobacco heating systems are NOT associated with an increased health risk?	Strongly agree	551	51.8	1.5
	Agree	266	25.0	1.3
	Neither agree nor disagree	120	11.3	1.0
	Disagree	65	6.1	0.7
	Strongly disagree	61	5.7	0.7
	**Total number of respondents**	1063	100.0	
To what extent do you agree with the statement that e-cigarettes/vapes are NOT associated with an increased health risk?	Strongly agree	594	55.9	1.5
	Agree	252	23.7	1.3
	Neither agree nor disagree	93	8.7	0.9
	Disagree	66	6.2	0.7
	Strongly disagree	58	5.5	0.7
	**Total number of respondents**	1063	1068.0	

Supplementary Table S22: Comparative analysis of the two groups of students according to the answers to the question "To what extent do you agree with the statement that classic cigarettes but in smaller quantities are NOT associated with an increased health risk?"

Group	Answers	Bulgarian students		Foreign students		P
		n	%	n	%	
All (n=1063)	Strongly agree	34	6.0	32	6.4	0.369
	Agree	31	5.5	39	7.8	
	Neither agree nor disagree	45	8.0	29	5.8	
	Disagree	131	23.3	109	21.8	
	Strongly disagree	322	57.2	291	58.2	
Men (n=417)	Strongly agree	19	8.5	11	5.7	0.211
	Agree	13	5.8	14	7.2	
	Neither agree nor disagree	17	7.6	6	3.1	
	Disagree	40	17.9	39	20.1	
	Strongly disagree	134	60.1	124	63.9	
Women (n=646)	Strongly agree	15	4.4	21	6.9	0.307
	Agree	18	5.3	25	8.2	
	Neither agree nor disagree	28	8.2	23	7.5	
	Disagree	91	26.8	70	22.9	
	Strongly disagree	188	55.3	167	54.6	

Supplementary Table S23: Comparative analysis of the two sexes according to the answers to the question "To what extent do you agree with the statement that classic cigarettes but in smaller quantities are NOT associated with an increased health risk?"

Group	Answers	Men		Women		P
		n	%	n	%	
All (n=1063)	Strongly agree	30	7.2	36	5.6	0.054
	Agree	27	6.5	43	6.7	
	Neither agree nor disagree	23	5.5	51	7.9	
	Disagree	79	18.9	161	24.9	
	Strongly disagree	258	61.9	355	55.0	
Bulgarian students (n=563)	Strongly agree	19	8.5	15	4.4	0.061
	Agree	13	5.8	18	5.3	
	Neither agree nor disagree	17	7.6	28	8.2	
	Disagree	40	17.9	91	26.8	
	Strongly disagree	134	60.1	188	55.3	
Foreign students (n=500)	Strongly agree	11	5.7	21	6.9	0.159
	Agree	14	7.2	25	8.2	
	Neither agree nor disagree	6	3.1	23	7.5	
	Disagree	39	20.1	70	22.9	
	Strongly disagree	124	63.9	167	54.6	

Supplementary Table S24: Comparative analysis of the two groups of students according to the answers to the question "To what extent do you agree with the statement that Tobacco heating systems are NOT associated with an increased health risk?"

Group	Answers	Bulgarian students		Foreign students		P
		n	%	n	%	
All (n=1063)	Strongly agree	31	5.5	30	6.0	0.303
	Agree	41	7.3	24	4.8	
	Neither agree nor disagree	70	12.4	50	10.0	
	Disagree	138	24.5	128	25.6	
	Strongly disagree	283	61.6	268	64.3	
Men (n=417)	Strongly agree	18	8.1	10	5.2	0.100
	Agree	16	7.2	7	3.6	
	Neither agree nor disagree	30	13.5	19	9.8	
	Disagree	45	20.2	55	28.4	
	Strongly disagree	114	51.1	103	53.1	
Women (n=646)	Strongly agree	13	3.8	20	6.5	0.310
	Agree	25	7.4	17	5.6	
	Neither agree nor disagree	40	11.8	31	10.1	
	Disagree	93	27.4	73	23.9	
	Strongly disagree	169	49.7	165	53.9	

Supplementary Table S25: Comparative analysis of the two sexes according to the answers to the question "To what extent do you agree with the statement that Tobacco heating systems are NOT associated with an increased health risk?"

Group	Answers	Men		Women		P
		n	%	n	%	
All (n=1063)	Strongly agree	28	6.7	33	5.1	0.742
	Agree	23	5.5	42	6.5	
	Neither agree nor disagree	49	11.8	71	11.0	
	Disagree	100	24.0	166	25.7	
	Strongly disagree	217	52.0	334	51.7	
Bulgarian students (n=563)	Strongly agree	18	8.1	13	3.8	0.108
	Agree	16	7.2	25	7.4	
	Neither agree nor disagree	30	13.5	40	11.8	
	Disagree	45	20.2	93	27.4	
	Strongly disagree	114	51.1	169	49.7	
Foreign students (n=500)	Strongly agree	10	5.2	20	6.5	0.707
	Agree	7	3.6	17	5.6	
	Neither agree nor disagree	19	9.8	31	10.1	
	Disagree	55	28.4	73	23.9	
	Strongly disagree	103	53.1	165	53.9	

Supplementary Table S26: Comparative analysis of the two groups of students according to the answers to the question "To what extent do you agree with the statement that E-cigarettes/vapes are NOT associated with an increased health risk?"

Group	Answers	Bulgarian students		Foreign students		P
		n	%	n	%	
All (n=1063)	Strongly agree	36	6.4	30	6.0	0.694
	Agree	27	4.8	31	6.2	
	Neither agree nor disagree	53	9.4	40	8.0	
	Disagree	128	22.7	124	24.8	
	Strongly disagree	319	56.7	275	55.0	
Men (n=417)	Strongly agree	20	9.0	8	4.1	**0.046**
	Agree	13	5.8	14	7.2	0.562
	Neither agree nor disagree	21	9.4	16	8.2	0.667
	Disagree	40	17.9	55	28.4	**0.011**
	Strongly disagree	129	57.8	101	52.1	0.344
Women (n=646)	Strongly agree	16	4.7	22	7.2	0.465
	Agree	14	4.1	17	5.6	
	Neither agree nor disagree	32	9.4	24	7.8	
	Disagree	88	25.9	69	22.5	
	Strongly disagree	190	55.9	174	56.9	

Supplementary Table S27: Comparative analysis of the two sexes according to the answers to the question "To what extent do you agree with the statement that E-cigarettes/vapes are NOT associated with an increased health risk?"

Group	Answers	Men		Women		P
		n	%	n	%	
All (n=1063)	Strongly agree	28	6.7	38	5.9	0.745
	Agree	27	6.5	31	4.8	
	Neither agree nor disagree	37	8.9	56	8.7	
	Disagree	95	22.8	157	24.3	
	Strongly disagree	230	55.2	364	56.3	
Bulgarian students (n=563)	Strongly agree	20	9.0	16	4.7	0.074
	Agree	13	5.8	14	4.1	
	Neither agree nor disagree	21	9.4	32	9.4	
	Disagree	40	17.9	88	25.9	
	Strongly disagree	129	57.8	190	55.9	
Foreign students (n=500)	Strongly agree	8	4.1	22	7.2	0.339
	Agree	14	7.2	17	5.6	
	Neither agree nor disagree	16	8.2	24	7.8	
	Disagree	55	28.4	69	22.5	
	Strongly disagree	101	52.1	174	56.9	

Supplementary Table S28: Analysis of the relationship between the answers to the questions "To what extent do you agree with the statement that Classic cigarettes but in smaller quantities are NOT associated with an increased health risk?", "To what extent do you agree with the statement that tobacco heating systems are NOT associated with an increased health risk?" and "To what extent do you agree with the statement that E-cigarettes/vapes are NOT associated with an increased health risk?" and whether the respondents were smokers, non-smokers or former smokers

Questions	Answers	Frequency	Smokers (n=209)	Non-smokers (n=804)	Former smokers (n=50)
To what extent do you agree with the statement that classic cigarettes, but in smaller quantities, are NOT associated with an increased health risk? (p<0.001)	Strongly agree or Agree	n	41	89	6
		%	19.6[ac]	11.1[b]	12.0[bc]
	Neither agree nor disagree	n	23	46	5
		%	11.0[a]	5.7[bc]	10.0[ac]
	Disagree or Strongly disagree	n	145	669	39
		%	69.4[ac]	83.2[b]	78.0[bc]
To what extent do you agree with the statement that Tobacco heating systems are NOT associated with an increased health risk? (p=0.016)	Strongly agree or Agree	n	39	82	5
		%	18.7[ac]	10.2[b]	10.0[bc]
	Neither agree nor disagree	n	26	90	4
		%	12.4[a]	11.2[a]	8.0[a]
	Disagree or Strongly disagree	n	144	632	41
		%	68.9[ac]	78.6[b]	82.0[bc]
To what extent do you agree with the statement that e-cigarettes/vapes are NOT associated with an increased health risk? (p=0.014)	Strongly agree or Agree	n	33	86	5
		%	15.8[ac]	10.7[b]	10.0[bc]
	Neither agree nor disagree	n	27	60	6
		%	12.9[a]	7.5[bc]	12.0[ac]
	Disagree or Strongly disagree	n	149	658	39
		%	71.3[ac]	81.8[b]	78.0[bc]

* Same letters in the columns designate a lack of a statistically significant difference, while different letters designate the presence of such a difference (p<0.05)

Supplementary Table S29: Comparative analysis of the two groups of students (smokers only) according to the answers to the question „What effect do you believe smoking has exerted on your health?”

Group	Answers	Bulgarian students, smokers		Foreign students smokers		P
		n	%	n	%	
All (n=209)	Definitely negative	28	20,1	24	34,3	0,215
	Mostly negative	53	38,1	23	32,9	
	Neither negative nor positive	49	35,3	21	30,0	
	Mostly positive	8	5,8	2	2,9	
	Definitely positive	1	0,7	0	0,0	
Men (n=91)	Definitely negative	14	24,6	14	41,2	0,520
	Mostly negative	18	31,6	9	26,5	
	Neither negative nor positive	20	35,1	10	29,4	
	Mostly positive	4	7,0	1	2,9	
	Definitely positive	1	1,8	0	0,0	
Women (n=118)	Definitely negative	14	17,1	10	27,8	0,639
	Mostly negative	35	42,7	14	38,9	
	Neither negative nor positive	29	35,4	11	30,6	
	Mostly positive	4	4,9	1	2,8	
	Definitely positive	0	0,0	0	0,0	

Supplementary Table S30: Comparative analysis of the two sexes (smokers only) according to the answers to the question „What effect do you believe smoking has exerted on your health?"

Group	Answers	Men		Women		P
		n	%	n	%	
All (n=209)	Definitely negative	28	30,8	24	20,3	0,192
	Mostly negative	27	29,7	49	41,5	
	Neither negative nor positive	30	33,0	40	33,9	
	Mostly positive	5	5,5	5	4,2	
	Definitely positive	1	1,1	0	0,0	
Bulgarian students, smokers (n=139)	Definitely negative	14	24,6	14	17,1	0,431
	Mostly negative	18	31,6	35	42,7	
	Neither negative nor positive	20	35,1	29	35,4	
	Mostly positive	4	7,0	4	4,9	
	Definitely positive	1	1,8	0	0,0	
Foreign students smokers (n=70)	Definitely negative	14	41,2	10	27,8	0,662
	Mostly negative	9	26,5	14	38,9	
	Neither negative nor positive	10	29,4	11	30,6	
	Mostly positive	1	2,9	1	2,8	
	Definitely positive	0	0,0	0	0,0	
